# Conservative Management of Asymptomatic Pneumoperitoneum; Report of Two Cases

**Published:** 2019-01-03

**Authors:** Leila Alizadeh, Mahdieh Shakeri-Darzekonani, Amin Sadrazar, Masoud Nouri-Vaskeh, Sedigheh Basirjafari

**Affiliations:** 1 Liver and Gastrointestinal Diseases Research Center, Tabriz University of Medical Sciences, Tabriz, Iran; 2 Connective Tissue Diseases Research Center, Tabriz University of Medical Sciences, Tabriz, Iran; 3 Medical Philosophy and History Research Center, Tabriz University of Medical Sciences, Tabriz, Iran; 4 Department of Radiology, Tabriz University of Medical Sciences, Tabriz, Iran

**Keywords:** Peptic ulcer perforation, pneumoperitoneum, proton pump inhibitors, conservative treatment

## Abstract

Peptic ulcer disease is a common gastrointestinal disorder, the prevalence of which has reduced in recent years due to effective new treatments. Peptic ulcer perforation is an emergent life-threatening condition that causes pneumoperitoneum and septic shock. It often requires surgical procedures. We describe two cases of peptic ulcer perforation with only mild discomfort on the epigastric region since several months before. The patients were treated with a high dose proton pump inhibitor and conservative treatment without surgical procedures. Weekly follow up of the cases showed that the clinical condition of patients remained stable without any new signs and symptoms. This report shows that noninvasive treatment alone can be effective in some cases with mild symptoms.

## 1. Introduction:

Peptic ulcer diseases (PUD) is one of the common gastroduodenal disorders that affects about 4 million people per year (1). Gastrointestinal (GI) perforation is a life-threatening complication of PUD (2). It causes about 5% of abdominal emergencies with up to 30% mortality and up to 50% morbidity rates (3, 4). Due to *H. pylori *eradication by effective antibiotic therapy and widespread use of protein pump inhibitors (PPIs), the prevalence of PUD has decreased. However, the life-threatening complications of PUD have not decreased (5). Peptic ulcer perforation presents as an acute abdomen with peritonitis, which can lead to septic shock and death. Radiological examination has a basic role in diagnosis of gastric ulcer perforation (6). Medical therapy, endoscopic and other non-surgical managements have decreased the role of surgery; nevertheless, the prevalence of peptic ulcer perforation is in a stable range and surgery has an important role in its treatment (7). However, due to the risk of complications after peptic ulcer perforation surgeries, sometimes other modalities other than open surgery should be considered (8). In the present paper, we report two asymptomatic pneumoperitoneum cases due to perforated peptic ulcer, which were treated with high dose PPI and conservative treatment without surgical procedures.

## 2. Case presentation:

***2.1. Case 1***

**Figure 1:**
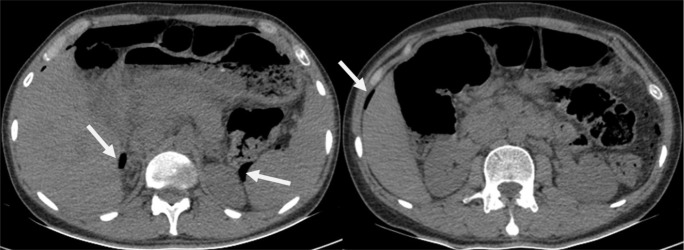
Pneumoperitoneum in pararenal space and right perihepatic space

A 34-year-old man with a 2-year history of peptic ulcer was admitted to the gastrointestinal (GI) ward in a tertiary referral hospital. The patient’s chief complaints were one episode of hematemesis 4 days ago and persistent mild abdominal pain in the epigastric region. The patient was addicted to 2.5 mg methadone per day. Vital signs were in normal ranges. Physical examination was normal and did not demonstrate any tenderness and guarding in the abdomen. Upper GI endoscopy was performed after 12 hours of fasting. A clean base gastric ulcer in the gastric outlet with gastric outlet obstruction pattern and grade A esophagitis were seen in the upper GI endoscopy. The baseline laboratory analysis revealed the following: white blood cell (WBC) Count: 6100/µL (Segment: 60.4%), Hemoglobin: 15.6g/dl; Platelet Count: 176000/µL; LDH: 260 IU/mL and, other lab tests including liver tests, creatinine, and blood urea nitrogen, were within the normal range. Serial laboratory test measurements did not show any out of range changes. Non-contrast abdominal computed tomography (CT) revealed pneumoperitoneum around the stomach and liver (Figure 1). CT scan with contrast revealed hydro pneumoperitoneum at porta hepatis and aortocaval regions. The consultant surgeon recommended follow up by endoscopy and antibiotic therapy. The patient was treated with high dose pantoprazole, hydration and bowel rest and intravenous ceftriaxone and, metronidazole. During hospitalization, abdominal pain resolved and no abdominal tenderness and guarding developed. Finally, he was discharged in good health with high dose oral pantoprazole, metronidazole and cefixime, and was also advised to refer to the GI clinic after a week. Four-month weekly follow up showed no abdominal symptoms and normal quality of life.

***2.2. Case 2***

**Figure 2:**
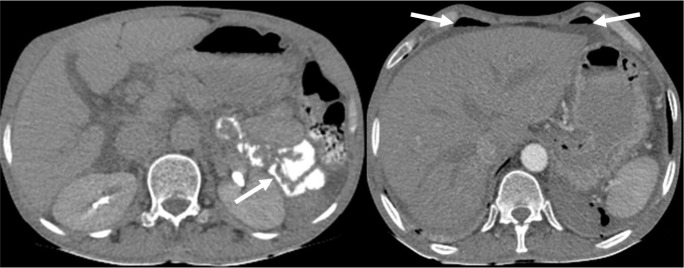
Oral and intravenous contrast abdominal computed tomography scan. The leak of the contrast material from gastrointestinal tract and its extension to the left pararenal space was seen. Ascites and pneumoperitoneum were seen in subhepatic space

A 52-year-old man with a history of dyspepsia from 6-8 months before with chief complaint of three hematemesis episodes 3 days before was admitted to the GI ward. He had no history of melena, hematochezia, dyspnea, fatigue, syncope and orthostatic dizziness. He had no history of PUD and had not reported taking non-steroidal anti-inflammatory drugs (NSAIDs), PPIs or histamine receptor antagonists. He was not an alcoholic or smoker. He was addicted to opiates. The patient’s familial history was negative. Physical examination on admission revealed stable vital signs and normal abdominal examination. Rectal examination was normal and no visible hemorrhoid was observed. His endoscopy showed scar and ulcer in the duodenal bulb and, duodenal bulb deformity due to a chronic ulcer. Abdominal CT scan revealed a pneumoperitoneum (Figure 2). Consultant surgeon recommended follow up. The baseline laboratory analysis during admission revealed the following: WBC Count: 7200/µL (Segment: 63.8%); Hemoglobin: 13.0g/dl; Platelet Count: 228000/µL; Creatinine: 1.03mg/dl; ESR: 9 mm/hr; CRP: 40 mg/l. Liver function tests and coagulation panel were within normal limits. During hospitalization, there were no out of rage changes in laboratory findings. He was treated with intravenous ceftriaxone and, metronidazole and also high dose pantoprazole. After 10 days, the patient was discharged from the hospital with oral pantoprazole, metronidazole and cefixime. Five-month weekly follow up showed that during that time there were only a few problems due to the dyspepsia he already had.

## 3. Discussion

Perforated peptic ulcer is a condition, which causes the leakage of gas and gastroduodenal content into the peritoneal cavity. Peptic ulcer perforation mostly affects the anterior wall of the duodenum, and the antral and lesser curvature of stomach (9). An accurate history, physical examination, and CT scan as a more sensitive method are required for diagnosis of pneumoperitoneum (10). The patient’s history and endoscopy observations along with imaging findings most probably help distinguish the free air in the abdomen cavity due to peptic ulcer perforation. When there is no sign of peritonitis and the patient's general condition is well, conservative treatment should be considered (11, 12). Multidetector CT scan is more helpful for detecting the origin of GI tract perforation (13). Proximal GI perforation is probable when free air is present just around the liver, stomach, duodenum and not in the pelvis (14). In these cases abdominal CT scan should be performed. In case 1, on CT scan, incidentally, pneumoperitoneum was found around the stomach, which was probably related to gastric perforation. In case 2, CT scan showed pneumoperitoneum around the duodenum, which was likely related to duodenal perforation. Patients with pneumoperitoneum in the upper abdomen (such as the two cases in this report), who have mild symptoms, stable hemodynamics and no signs of acute abdomen might have micro perforation in their stomach (like case 1) and duodenum (like case 2), in which case they should initiate conservative therapy with anti-ulcer agents such as PPI, H_2_ blocker, and intravenously administered antibiotics. The patient should be followed carefully (15). The follow up of these two cases showed that their condition remained stable.

In conclusion, we reported two pneumoperitoneum cases due to peptic ulcer perforation with mild symptoms and stable conditions, which were managed without the need for surgical procedures. More similar cases and further evaluations are needed for better comprehension of the outcome of conservative treatment in asymptomatic peptic ulcer perforations.
